# 

**Published:** 1891-03

**Authors:** 


Bristol Medico-Chirurgical Journal.

MARCH, 1891.

CONTENTS.

| | |
|---|---|
| Original Papers: | Page |
| Puerperal Eclampsia. By J. G. Swayne, M.D. ... ... ... ... .. ... | 1 |
| Case of Poisoning by Rhus Venenata. By Arthur W. Prichard, M.R.C.S. ... ... ... ... ... ... ... ... ... ... ... ... ... ... | 22 |
| A Case of Laryngeal Tuberculosis. By P. Watson Williams, M.B. Lond. ... ... ... ... ... . ... ... ... ... ... ... ... ... ... | 27 |
| Progress of the Medical Sciences ... ... ... ... ... ... ... ... | 31 |
| Reviews of Books ... ... ... ... ... ... ... ... ... ... ... ... ... ... | 50 |
| Notes on Preparations for the Sick ... ... ... ... ... ... ... ... | 61 |
| Meetings ... ... ... ... ... ... ... ... ... ... ... ... ... ... ... | 63 |
| Library ... ... ... ... ... ... ... ... ... ... ... ... ... ... ... | 66 |
| Obituary ... ... ... ... ... ... ... ... ... ... ... ... ... ... ... | 67 |
| Royal College of Physicians of Edinburgh ... ... ... ... ... ... | 70 |
| Scraps ... ... ... ... ... ... ... ... ... ... ... ... ... ... ... | 71 |

*Cases for binding, price 7d. each, may be had of the Publisher,*

J. W. Arrowsmith, *Quay Street, Bristol.*

11

147

### THE BRISTOL

### Medico-Chirurgical Journal.

A JOURNAL OF THE MEDICAL SCIENCES FOR THE WEST OF ENGLAND AND SOUTH WALES.

*PUBLISHED UNDER THE AUSPICES OF*

*THE BRISTOL MEDICO-CHIRURGICAL SOCIETY.*

EDITOR:

J. GREIG SMITH, M.A., F.R.S.E.

ASSOCIATED WITH

BARCLAY J. BARON, M.B., C.M. F. R. CROSS, M.B.

J. MICHELL CLARKE, M.B., M.A. W. H. HARSANT.

ASSISTANT-EDITOR:

L. M. GRIFFITHS.

"Scire est nescire, nisi id me
Scire alius sciret."

*VOL. IX.*

BRISTOL: J. W. ARROWSMITH;
LONDON: J. & A. CHURCHILL, 11 NEW BURLINGTON STREET.
1891.

L. H.

R 31
B 8

### CONTENTS OF VOLUME IX.

Page

Puerperal Eclampsia. By J. G. SWAYNE, M.D. ... ... ... ... 1

Case of Poisoning by Rhus Venenata. By ARTHUR W. PRICHARD, M.R.C.S. Eng. ... ... ... ... ... ... ... ... ... ... ... 22

Case of Laryngeal Tuberculosis. By P. WATSON WILLIAMS, M.B. Lond. ... ... .. ... ... ... ... ... ... ... ... 27

On Modern Methods of Diagnosis in Gastric Affections. By J. MICHELL CLARKE, M.A., M.B. Cantab., M.R.C.P.... .. 73

Case of Septic Endocarditis with Cerebral Embolism. By F. H. EDGEWORTH, M.B., B.C., B.A. Cantab., B.Sc. Lond. ... 89

Chloroform *versus* Ether. By W. H. HARSANT, F.R.C.S. Eng.... 145

Cases of Septic Thrombosis of the Lateral Sinus. By CHARLES F. PICKERING, F.R.C.S. Eng. ... ... ... ... ... ... ... 155

Case of Tumour of the Pons. By P. WATSON WILLIAMS, M.B. Lond. ... .. ... ... ... ... ... ... ... ... ... 163

Buxton: Its Baths and Climate. By ALFRED J. H. CRESPI, M.R.C.S. Eng., M.R.C.P.I. ... ... ... ... ... ... ... ... 167

Medical Progress, Local and General. By F. R. CROSS, M.B. Lond., F.R.C.S. Eng. .. ... ... ... ... ... ... ... ... ... ... 241

Epilepsy Treated by Sulphonal. By GILBERT A. BANNATYNE, M.D., C.M. Glasg. ... ... ... ... ... ... ... ... ... ... 261

Page

HEALTH-RESORTS IN THE WEST OF ENGLAND AND SOUTH WALES
Malvern. By WALTER TYRRELL, M.R.C.S. Eng., L.S.A. ... ... 267

PROGRESS OF THE MEDICAL SCIENCES ... ... ... 31, 94, 184, 278

REVIEWS OF BOOKS ... ... ... ... ... ... ... 50, 119, 204, 299

NOTES ON PREPARATIONS FOR THE SICK ... ... ... 61, 137, 213, 308

MEETINGS OF THE BRISTOL MEDICO-CHIRURGICAL SOCIETY ... ... ... ... ... ... ... ... ... 63, 140, 311

THE LIBRARY OF THE BRISTOL MEDICO-CHIRURGICAL SOCIETY ... ... ... ... ... ... ... ... ... 66, 215, 313

OBITUARY ... ... ... ... .. ... ... ... ... ... ... ... ... ... 67

ROYAL COLLEGE OF PHYSICIANS OF EDINBURGH ... ... ... ... ... 70

VIRCHOW TESTIMONIAL FUND ... ... ... ... ... ... ... ... ... 142

SCRAPS ... ... ... ... ... ... ... ... ... ... 71, 143, 238, 319

### The Library of the Bristol Medico-Chirurgical Society.

The Committee, taking into consideration an influential request from a large number of the members of this Society, recently resolved to establish a reference library and reading-room. The formation of the Literary and Philosophic Club at 28 Berkeley Square, which had for one of its principal objects the accommodation under one roof of the scientific societies of the neighbourhood, offered one of its best rooms at a moderate rent to the Medico-Chirurgical Society. This offer was accepted; and so, amidst congenial surroundings, and with comfortable accommodation, the room was formally opened on January 5th by Mr. S. H. Swayne, the President, who was supported by several Past-Presidents and other influential members of the Society. Short speeches were made by the President, Mr. Augustin Prichard, Dr. J. G. Swayne, Dr. Shingleton Smith, and Mr. Nelson Dobson. Through the liberality of some members and others, and by the aid of the *Journal*, a considerable amount of literature is now at the service of those who belong to the Society; and with such an attraction the

Society should, within a very short time, find its numbers considerably increased.

There are now in the library over a thousand volumes, and 79 periodicals. None of these works are intended for general circulation; but members of the *Journal* staff may take out for a period not exceeding one week any periodical except the current number. Members have thus the advantage of knowing that practically they will find in the room any publication the Society possesses.

Amongst the books will be found many of the recent larger works of reference. The periodical literature, which comes from all parts of the world, is represented by 79 journals. Of these, 2 are published three times a week, 1 twice a week, 20 weekly, 7 fortnightly, 31 monthly, 6 quarterly, 2 half-yearly, and 10 yearly.

Mr. L. M. Griffiths, who has been appointed Honorary Librarian, will be glad to receive any books for the Library. Long sets of periodicals, transactions, and hospital reports, and works in several volumes, will be specially acceptable.

### Bristol Medico-Chirurgical Society.

### President.

S. H. SWAYNE.

### President-Elect.

F. R. CROSS, M.B. Lond.

### Honorary Secretary.

G. MUNRO SMITH.

### Committee.

*Past Presidents.*

R. W. COE.
AUGUSTIN PRICHARD, M.D. Berlin.
J. G. SWAYNE, M.D. Lond.
E. LONG FOX, M.D. Oxon.
JAMES G. DAVEY, M.D. St. And.
W. JOHNSTONE FYFFE, M.D. Dub.
G. F. BURDER, M.D. Aberd.
W. H. SPENCER, M.D., M.A. Cantab.
F. POOLE LANSDOWN.
L. M. GRIFFITHS.
R. SHINGLETON SMITH, M.D., B.Sc. Lond.
NELSON C. DOBSON.

A. J. HARRISON, M.B. Lond.
W. H. HARSANT.
ARTHUR W. PRICHARD.
J. E. SHAW, M.B., C.M. Ed.
E. MARKHAM SKERRITT, M.D., B.S., B.A. Lond.
J. GREIG SMITH, M.B., C.M., M.A. Aberd., F.R.S.E.

Medical Men wishing to join the Society are requested to communicate with the Honorary Secretary, G. MUNRO SMITH, 27 All Saints Road, Clifton, Bristol. The Annual Subscription is 10/6.

---

*Extracts from Journal By-laws.*

4.—The *Journal* shall be delivered free to Subscribers and also to Members of the Society whose subscriptions are not in arrear.

6.—The Annual Subscription to the *Journal* for those who pay before the 1st of July shall be 5/-, post free, or 6/- if paid after that date.

---

Communications intended for insertion in the *Journal* should be sent to the Editor, J. GREIG SMITH, M.A., F.R.S.E., 16 Victoria Square, Clifton, Bristol.

Books for Review, Exchange Journals, and communications referring to the delivery of the *Journal* should be sent to the Assistant-Editor, L. M. GRIFFITHS, 9 Gordon Road, Clifton, Bristol, to whom Subscriptions are to be paid *in advance*.

Authors requiring Reprints of their Papers should communicate with the Publisher.

Cases for Binding Vols. I.–VIII. are now ready, and may be obtained, price 7*d*. each (postage 2*d*. extra), upon application to J. W. ARROWSMITH, Quay Street, Bristol; or the *Journal* will be bound, including Case, for 1/3 per volume.

### Bristol Medico-Chirurgical Society.

---

### PAST PRESIDENTS.

1874—75. FREDERICK BRITTAN, M.D., B.A. Dub.

1875—76. ROBERT WILLIAM COE.

1876—77. SAMUEL MARTYN, M.D. Ed.

1877—78. AUGUSTIN PRICHARD, M.D. Berlin.

1878—79. HENRY EDWARD FRIPP, M.D. Würzburg.

1879—80. WILLIAM MICHELL CLARKE.

1880—81. JOSEPH GRIFFITHS SWAYNE, M.D. Lond.

1881—82. EDWARD LONG FOX, M.D. Oxon.

1882—83. JAMES GEORGE DAVEY, M.D. St. And.

1883—84. WILLIAM JOHNSTONE FYFFE, M.D. Dub.

1884—85. GEORGE FORSTER BURDER, M.D. Aberd.

1885—86. WILLIAM HENRY SPENCER, M.D., M.A. Cantab.

1886—87. FRANCIS POOLE LANSDOWN.

1887—88. LEMUEL MATTHEWS GRIFFITHS.

1888—89. ROBERT SHINGLETON SMITH, M.D., B.Sc. Lond.

1889—90. NELSON CONGREVE DOBSON.

1891.

---

### LIST OF SUBSCRIBERS

TO THE

### Bristol Medico-Chirurgical Journal.

---

*Those marked * are Members of the Society.*

| *Name.* | *Residence.* |
| --- | --- |
| Abercrombie, John, M.D. Cantab. | 23 Upper Wimpole Street, London, W. |
| Ackland, J. McKno | 24 Southernhay, Exeter. |
| *Ackland, W. R. | 5 Rodney Place, Clifton. |
| Adams, George | 11 Westbourne Place, Clifton. |
| *Adams, John Barfield | 110 Cotham Road, Bristol. |
| Adye, W. John A. | Church House, Bradford-on-Avon. |
| Alexander, James, M.D., M.Ch., B.A. Dub. | Bishop's Place, Paignton. |
| *Alford, George Ernest | 5 Royal Crescent, Weston-super-Mare |
| Alford, Henry | 9 Mountlands, Taunton. |
| Alford, Henry J., M.D. Lond. | 2 Marlborough Terrace, Taunton. |
| Appleton, Henry, M.D. Aberd. | Poulton Lodge, Stoke Bishop. |
| Arthur, John | Hilton, Llandaff. |
| *Atchley, George F., M.B. Lond. | Tyndall's Park, Bristol. |
| *Aveline, H. T. S. | Asylum, Stapleton, Gloucestershire. |
| | |
| Baker, A. de W. | 2 Lawn Terrace, Dawlish. |
| *Barclay, W. M. | Queen's Road, Clifton. |
| Baretti, T. G. L'Enardi | 49 Royal York Crescent, Clifton |
| *Baron, Barclay J., M.B., C.M. Ed. | 16 White Ladies Road, Clifton. |
| *Basset, Walter | 24 Stow Hill, Newport, Mon. |
| Batten, Rayner W., M.D. Lond. | 1 Brunswick Square, Gloucester |
| Batten, W. Smith | Curzon House, Calne. |
| Beale, Lionel S., M.B. Lond., F.R.S. | 61 Grosvenor Street, London, W |
| *Beddoe, John, M.D. Ed., B.A. Lond., F.R.S. | The Manor House, Clifton. |
| Benham, Harry A., M.D., C.M. Aberd. | Asylum, Stapleton, Gloucestershire. |
| Bennett, W. S. | Escot, Penzance. |
| *Benson, A. H. | Wrington. |

### LIST OF SUBSCRIBERS.

| | |
|---|---|
| Berry, F. C., M.D., B.Ch., B.A. Dub. | Lyn Cottage, Lynton. |
| *Blacker, A. E. | Richmond Hill Avenue, Clifton. |
| Blacker, E. | Midsomer Norton. |
| *Blagg, Arthur F. | 6 West Mall, Clifton. |
| Blomfield, Arthur G., M.D. Aberd. | 34 West Southernhay, Exeter. |
| Bloxam, G. E. | Cambridge House, Lower Weston, Bath. |
| *Board, Edmund C. | 11 Caledonia Place, Clifton. |
| *Boyce, James G. | 10 Redcliff Parade, Bristol. |
| Boys, Arthur Henry | Chequer Street, St. Alban's. |
| Brabazon, A. B., M.D. Aberd. | 12 Darlington Street, Bath. |
| Bright, Eustace F., M.D. Lond. | Exeter Road, Bournemouth. |
| Bright, J. A. | Glastonbury. |
| *Broom, John, M.D., Ch.D., Obst.D. Brussels | St. Paul's Road, Clifton. |
| Brown, G. Arthur | Tredegar. |
| Brown, William, M.D., C.M. Glasg. | Fishponds, Gloucestershire. |
| Browne, A. E. Newbury | Burbage, Wiltshire. |
| Browne, John, M.D. Ed. | Dundalk. |
| Browne, G. Henry | The Hermitage, Brynmawr, Breconshire. |
| Browne, J. Walton, M.D., B.A. Roy. Univ. I. | 10 College Square North, Belfast. |
| Browne, Lennox | 36 Weymouth Street, London, W. |
| *Burder, George F., M.D. Aberd. | 63 Oakfield Road, Clifton. |
| Burges, R. E., M.D., M.Ch., B.A. Roy. Univ. I. | 3 Egerton Terrace, Hoole, Chester. |
| Burroughs, E. F. H. | 3 Elgin Park, Bristol. |
| *Bush, J. Paul | 13 Lansdown Place, Clifton. |
| Buzzard, Thomas, M.D. Lond. | 74 Grosvenor Street, London, W. |
| | |
| *Calder, F., M.B. Lond. | 14 Belle Vue, Cotham, Bristol. |
| *Cameron, John, M.B., C.M. Glasg. | 63 Cotham Road, Bristol. |
| Cann, Francis M. | Sefton House, Dawlish. |
| *Carr, Alexander | Wells Road, Bristol. |
| Carter, R. W., M.D. Calcutta | 13 Gloucester Row, Weymouth. |
| *Carter, W. F. | 48 Coronation Road, Bristol. |
| Cave, Edward J., M.D. Lond. | Crewkerne. |
| Clapp, W. J. | Earl Street, Penarth. |
| Clark, Thomas | Blair, Minehead, Somersetshire. |
| Clarke, Arthur, B.A. Lond. | Street, Somersetshire. |
| *Clarke, John Michell, M.B., M.A. Cantab. | Clifton Road, Clifton. |
| Clay, Challoner | Manor House, Fovant. |
| Clay, R. Hogarth, M.D. Ed. | 4 Windsor Villas, Plymouth. |
| Coathupe, Edwin W. | 7 Clifton Park, Clifton. |
| Cockey, Edmund | West Lodge, Frome. |
| *Coe, R. W. | 7 Pembroke Road, Clifton. |
| Coker, T. V. | 2 Chesterfield Place, Clifton. |
| Cole, Thomas, M.D. Lond. | 18 Circus, Bath. |
| *Collins, H. W. | Wrington, Somersetshire. |
| Colman, Thomas J., M.D. Ed. | Elmgrove Road, Bristol. |
| Colmer, Ptolemy S. H., M.D. Dur. | South Street House, Yeovil. |
| Cooke, A. S. | Badbrook House, Stroud. |
| *Corbould, George G. | 22 Berkeley Square, Bristol. |
| *Cotton, William, M.B., C.M. Ed. | 227 Gloucester Road, Bristol. |
| Cousins, J. Ward, M.D. Lond. | Kent Road, Southsea. |
| Cowan, F. S. | 27 Queen Square, Bath. |
| Cox, William I. | Hawkesbury-Upton. |

### LIST OF SUBSCRIBERS.

| | |
|---|---|
| Craddock, Samuel | 1 Green Park, Bath. |
| Crespi, Alfred J. H. | Poole Road, Wimborne. |
| *Cross, F. Richardson, M.B. Lond. | Richmond Park Road, Clifton. |
| *Crossman, Edward, M.D. Dur. | Hambrook, Gloucestershire. |
| | |
| *Dacre, John | St. Paul's Road, Clifton. |
| Daniel, T. P. | Beaminster, Dorsetshire. |
| *Davey, James G., M.D. St. And. | 31 Avonmore Road, London, W. |
| *Davies, D. S., M.B. Lond. | 60 Oakfield Road, Clifton. |
| Davies, Ebenezer | Brunswick House, Swansea. |
| Davies, E. Cluneglas | Pontfaen, Lampeter, Cardiganshire. |
| Davies, H. N. | Porth, Glamorganshire. |
| Davis, Robert | 14 St. James's Square, Bath. |
| Davis, Theodore, M.D. Lond. | Beachcroft, Clevedon. |
| Davy, Henry, M.D. Lond. | Southernhay House, Exeter. |
| Day, Percy Howard | Stalmine, Poulton-le-Fyldc. |
| Deas, P. Maury, M.B., M.S. Lond. | Wonford House, Exeter. |
| Dening, Edwin | Stow-on-the-Wold. |
| *Devis, Harry F. | Wells Road, Bristol. |
| *Dew, Henry R. | 25 Berkeley Square, Bristol. |
| *Dobson, Nelson C. | 27 Victoria Square, Clifton. |
| Domville, Edward J. | 44 Southernhay West, Exeter. |
| *Dowson, W., M.B., B.C., M.A. Cantab. | Great George Street, Bristol. |
| Doyne, Robert W. | 70 Woodstock Road, Oxford. |
| Duffett, E. D. | 5 London Street, Swindon, Wiltshire. |
| Dunbar, Eliza L. Walker, M.D. Zürich | 9 Oakfield Road, Clifton. |
| *Duncan, William | Westbury-on-Trym. |
| | |
| Eadon, W. F. Bailey | Hambrook, Gloucestershire. |
| *Eager, Reginald, M.D. Lond. | Northwoods, Winterbourne. |
| Eddowes, Charles | Maddington, Devizes. |
| *Edgeworth, F. H., M.B., B.C., B.A. Cantab., B.Sc. Lond. | 1 Richmond Hill, Clifton. |
| Edwards, W. T., M.D. Lond. | Springfield House, Cardiff. |
| Elder, William | 7 Trelawney Road, Bristol. |
| *Elliott, Christopher, M.D., B.A. Dub. | 88 Pembroke Road, Clifton. |
| Ellis, Henry V., M.B., C.M. Aberd. | Ty Bryn, Reynoldstone. |
| Ellis, T. S. | 6 Clarence Street, Gloucester. |
| Ellis, W. Mc.D., M.D. Brussels | 7 Alexander Buildings, Bath. |
| Embleton, Dennis C. | St. Michael's Road, Bournemouth. |
| *Etches, W. R. | 13 Dowry Square, Clifton. |
| Evans, J. Fenton, M.B., C.M. Ed. | 6 Whitehall Yard, London, S.W. |
| Evans, W. G. | Beckington. |
| *Ewens, John | 14 White Ladies Road, Clifton. |
| | |
| Fairbanks, W., M.D., C.M. Ed. | Wells, Somersetshire. |
| *Fendick, R. George | St. Paul's Road, Clifton. |
| Fernie, James | Stratton St. Margaret. |
| Finch, Thomas, M.D. St. And. | Westville, St. Mary Church. |
| Fisher, Fred. B. | West Walk, Dorchester. |
| Flemming, C. E. S. | Freshford. |
| Ford, Joseph | Wedmore. |
| Forty, D. H. | Wotton-under-Edge. |

| | |
|---|---|
| Fox, Arthur E. W., M.B., C.M. Ed. | 16 Gay Street, Bath. |
| *Fox, Bonville B., M.D., M.A. Oxon. | Brislington. |
| Fox, Charles Henry, M.D. St. And. | Brislington. |
| *Fox, E. Long, M.D. Oxon. | Church House, Clifton. |
| Fox, John Alfred | Morrab Road, Penzance. |
| Fraser, Græme B. | Beach Road, Weston-super-Mare. |
| Freeman, Henry W. | 24 Circus, Bath. |
| Fry, John B. | Ashley Lodge, Esher. |
| Furse, Edwin | South Molton. |
| *Fyffe, W. Johnstone, M.D., B.A. Dub. | 2 Rodney Place, Clifton. |
| | |
| Gamble, C. H. | Barnstaple. |
| Gardiner, George | 1 Redcliff Hill, Bristol. |
| *Gibbs, Alfred N. Godby | 27 Chandos Road, Bristol. |
| Gill, John, M.D. Brussels | 31 Apsley Road, Clifton. |
| *Gloag, G. A. | 7 College Green, Bristol. |
| Goodden, Wyndham C. | 14 Berkeley Square, Bristol. |
| Goodridge, Henry F. A., M.D. Lond. | 10 Brock Street, Bath. |
| Goss, T. Biddulph | 1 Circus, Bath. |
| Gould, A. Pearce, M.B., M.S. Lond. | 10 Queen Anne Street, London, W. |
| Grace, Alfred | Chipping Sodbury. |
| Grace, Edward Mills | Thornbury, Gloucestershire. |
| Grace, Henry | Kingswood, Bristol. |
| Grace, W. G. | 57 Stapleton Road, Bristol. |
| Granville, J. Mortimer, M.D. St. And. | 14 Hanover Square, London, W. |
| Gray, F. A. | Ottery St. Mary. |
| *Griffiths, L. M. | 9 Gordon Road, Clifton. |
| Grigor, Macadam | Alexandra Avenue, London, S.W. |
| Grote, G. W., M.B. Toronto | 24 St. Andrew's Crescent, Cardiff. |
| Groves, J., M.B., B.A. Lond. | Carisbrooke. |
| Guinness, T. A. | Bampton, Devonshire. |
| | |
| *Hailes, Clements D. G., M.D., C.M. Ed. | 9 King's Parade, Clifton. |
| *Haines, A. H. | 8 Cotham Road, Bristol. |
| *Hale, Edmund T. | The Grange, Chew Magna. |
| Handfield-Jones, Montagu, M.D. Lond. | 24 Montagu Square, London, W. |
| Hardwick, A., M.B. Dur. | New Quay, Cornwall. |
| Harper, Joseph | Bear Street, Barnstaple. |
| Harris, John H. | 30 Church Street, Modbury. |
| *Harrison, A. J., M.B. Lond. | Guthrie Road, Clifton. |
| *Harsant, W. H. | 16 Pembroke Road, Clifton. |
| Hawkins, Cæsar F. | North Petherton. |
| Haydon, Edgar, M.B., C.M. Glasg. | The Laurels, Newton Abbot. |
| Hayman, Alfred G. | 1 Pembroke Road, Clifton. |
| Heane, W. C. | Cinderford. |
| Hearder, G. J., M.D. St. And. | Joint Counties Asylum, Carmarthen. |
| *Heaven, John C. | 2 Queen Square, Bristol. |
| *Herapath, C. K. C. | 11 Brunswick Square, Bristol. |
| Herringham, W. P., M.D., B.A. Oxon. | 13 Upper Wimpole Street, London, W. |
| *Hetling, H. E. | Stapleton Road, Bristol. |
| Hichens, James Stacey | 17 Green Lane, Redruth. |

| | |
|---|---|
| Hill, Hedley | Carmarthenshire Infirmary, Carmarthen. |
| Hills, Frederic F. | 164 Stapleton Road, Bristol. |
| Hinton, Joseph | The Chestnuts, Warminster. |
| Hoblyn, Francis Parker | 10 Bladud Buildings, Bath. |
| Hopkins, H. Culliford | 4 Gay Street, Bath. |
| Horsfall, John, M.A. Oxon. | Meyrick Road, Bournemouth. |
| Hospital, Bristol General (*Sec.*, W. Thwaites). | |
| Hospital, Taunton and Somerset (*House-Surgeon*, Basil W. Housman). | |
| Howells, William, M.B., C.M. Glasg. | Church House, Talgarth, Breconshire. |
| Howse, William | 8 London Street, Swindon, Wiltshire. |
| Howsin, E. Arthur, M.D. St. And. | Stroud. |
| Hudson, H. R. | 182 Commercial Road, Newport, Mon. |
| Huggard, W. R., M.D., M.Ch., M.A. Roy. Univ. I. | Davos-Platz, Switzerland. |
| Hunt, A. D. | Millbrook House, Chagford. |

| | |
|---|---|
| *Imlay, G. A., M.B., C.M. Aberd. | 1 Apsley Villas, Bristol. |
| Infirmary, Bristol Royal (*Librarian*, Walter J. Hill). | |
| Institution, Liverpool Medical (*Resident Librarian*, William Jones). | |

| | |
|---|---|
| Jackson, J. B., M.D., M.Ch. Roy. Univ. I. | 56 Lemon Street, Truro. |
| Jacobson, W. H. A., M.B., M.C., M.A. Oxon. | 66 Great Cumberland Place, London, W. |
| James, J. Bowen | Woodlands, Aberaman, Aberdare. |
| Jessop, Charles Moore | 98 Sutherland Avenue, London, W. |
| Jones, A. Orlando, M.D., C.M. Aberd. | Harrogate. |
| Jones, E. R. | Cilgynlle-fawr, New Quay, Cardiganshire. |
| Jones, Evan | Ty-Mawr, Aberdare. |
| Jones, W. R., M.D., C.M. Glasg. | Senny Bridge, Breconshire. |

| | |
|---|---|
| Kempe, Arthur W. | 9 Southernhay, Exeter. |
| Kendall, Bernard | Elm Grove, Tenby |
| King, Louis J. | 10 Russel Street, Bath. |
| Kinneir, R. | The Tower House, Malmesbury. |
| Kitson, F. P. | Beaminster, Dorsetshire. |

| | |
|---|---|
| Lace, F. | Royal United Hospital, Bath. |
| Laing, W. A. Gordon, M.D., C.M. Glasg. | Boutport Street, Barnstaple. |
| *Lane, Hugh | 11 Circus, Bath. |
| *Lansdown, F. Poole | 19 White Ladies Road, Clifton. |
| Lauwers, Dr. | Courtrai, Belgium. |
| *Lawrence, A. E. Aust, M.D., C.M. Aberd. | 19 Richmond Hill, Clifton. |
| *Lawrence, Henry | 19 Park Row, Bristol. |
| Lawrie, J. Macpherson, M.D., C.M. Glasg. | Greenhill, Weymouth. |
| Laxton, T. L. | Taunton Road, Bridgwater. |
| Leach, J. Comyns, M.D. Dur., B.Sc. Lond. | Sturminster Newton. |
| Leamon, Michael T. | 1 Bedford Villas, Tavistock. |
| *Lees, E. Leonard, M.D., C.M. Ed. | Redland Road, Bristol. |
| Leigh, W. W. | Treharris, Glamorganshire. |
| Lewellyn, John | Caerphilly. |
| Library, Bristol Museum and | Queen's Road, Bristol. |

### LIST OF SUBSCRIBERS.

| | |
|---|---|
| Library, Cheltenham ... ... ... ... ... | 5 Royal Crescent, Cheltenham. |
| *Lidderdale, James ... ... ... ... ... ... | Bourne End, Maidenhead. |
| Liddon, William, M.B. Lond. ... ... ... ... | Church Square, Taunton. |
| Lilley, G. Herbert, M.D. Brussels ... ... | H.M. Convict Prison, Portland. |
| Limbery, Thomas ... ... ... ... ... ... ... | 184 Commercial Road, Newport, Mon. |
| Lister, Sir Joseph, Bart., F.R.S. ... ... ... | 12 Park Crescent, London, N.W. |
| Lloyd, Walter E. ... ... ... ... ... ... | 60 York Road, Bristol. |
| *Logan, Frederic T. B. ... ... ... ... ... | Dean Lane, Bristol. |
| *Lowe, T. Pagan ... ... ... ... ... ... ... | 2 Belmont, Bath. |
| Luckham, L. S. ... ... ... ... ... ... ... | 16 Harcourt Terrace, Salisbury. |
| Lush, William G. Vawdrey, M.D. Lond. ... | 12 Frederick Place, Weymouth. |
| Macdonald, P. W., M.D., C.M. Aberd. ... | County Asylum, Dorchester. |
| *Maclean, Ewen J., M.B., C.M. Ed. ... ... | Children's Hospital, Bristol. |
| Maclean, J. Campbell, M.B., C.M Ed. ... | Swindon House, Swindon, Wiltshire. |
| Macready, J. ... ... ... ... ... ... ... ... | 51 Queen Anne Street, London, W. |
| *Maddison, W. T., M.D. Lond. ... ... ... ... | 39 Park Street, Bristol |
| Manning, H. J., B.A. Lond. ... ... ... ... | Laverstock House, Salisbury. |
| Marley, Henry F. ... ... ... ... ... ... | The Nook, Padstow, Cornwall. |
| Marsh, Charles J. ... ... ... ... ... ... | Yeovil. |
| Marsh, O. E. Bulwer ... ... ... ... ... | Ventnor House, Newport, Mon. |
| Marshall, Arthur L., M.B., B.A. Cantab. ... | 56 Rectory Road, London, N. |
| *Marshall, Henry, M.D. Ed., F.R.S.E. ... | 28 Caledonia Place, Clifton. |
| *Martin, E. F., M.B. Ed. ... ... ... ... ... | Victoria House, Weston-super-Mare. |
| Martin, Theodore ... ... ... ... ... ... | Temple Cloud. |
| Mason, S. B. ... ... ... ... ... ... ... ... | 12 Park Terrace, Pontypool. |
| Mason, William ... ... ... ... ... ... ... | Sedgemoor Cottage, St. Austell. |
| Masters, W. Hooper, M.D. St. And. ... ... | Parkstone. |
| *Matthews, Charles E. ... ... ... ... ... | 31 Pembroke Road, Clifton. |
| Mavrojanni, Dr. ... ... ... ... ... ... ... | Corfu, Greece. |
| *Mayor, T. O. ... ... ... ... ... ... ... ... | 40 Apsley Road, Clifton. |
| McNicoll, John ... ... ... ... ... ... ... | High Street, Poole. |
| Meeres, Edward E., M.D. Lond. ... ... ... | 1 St. Andrew's Terrace, Plymouth. |
| Meredith, John, M.D. Ed. ... ... ... ... | Wellington, Somersetshire. |
| Metford, J. Seymour ... ... ... ... ... | 34 West Mall, Clifton. |
| Michell, George A. ... ... ... ... ... ... | Redruth. |
| Milne, John, M.B., C.M. Aberd. ... ... ... | 97 Clarence Road, Bristol. |
| Milward, James, M.D. Brussels ... ... ... | 54 Charles Street, Cardiff. |
| Mole, Harold F. ... ... ... ... ... ... ... | 4 Aberdeen Road, Bristol. |
| Monckton, William ... ... ... ... ... ... | Portishead. |
| Morgan, Frederick ... ... ... ... ... ... | Uffculme. |
| Morgan, John ... ... ... ... ... ... ... ... | Langport, Somersetshire. |
| Morgan, William, M.D. Aberd. ... ... ... | 16 Edwards Terrace, Cardiff. |
| *Morton, C. A. ... ... ... ... ... ... ... ... | Zetland Road, Bristol. |
| Moynan, W. Arthur, M.D., M.Ch. Roy. Univ. I. | Penarth. |
| Munden, Charles ... ... ... ... ... ... ... | Ilminster. |
| Murray, William, M.B., C.M. Ed. ... ... | Staple Hill, Gloucestershire. |
| Napier, T. W. A., M.B., C.M. Aberd. ... ... | Church Street, Egremont, Cheshire |
| Nash, W. Gifford ... ... ... ... ... ... ... | South Devon Hospital, Plymouth. |
| *Nevill, W. N., M.B., B.Ch., B.A. Dub. ... | Southville, Bristol. |
| *Newman, Charles ... ... ... ... ... ... ... | 19 Portland Square, Bristol. |

### LIST OF SUBSCRIBERS.

| | |
|---|---|
| *Newnham, W. H. C., M.B., M.A. Cantab. ... | St. Paul's Road, Clifton |
| Newstead, James ... ... ... ... ... ... ... | 9 York Place, Clifton. |
| Newton, Charles John ... ... ... ... ... | Oriel Lodge, Cheltenham. |
| Nicholson, T. D., M.D., C.M. Ed. ... ... | 2 White Ladies Road, Clifton. |
| Norgate, R. H. ... ... ... ... ... ... ... | Totnes Road, Paignton. |
| *Norman, J. H. ... ... ... ... ... ... ... ... | Goodleigh, Winkleigh, Devonshire. |
| *Norton, J. A., M.D., C.M. Aberd. ... ... ... | 65 Redcliff Hill, Bristol. |
| | |
| *Ogilvy, Alexander, M.D., B.Ch., B.A. Dub. | Eye Hospital, Bristol. |
| Ollerhead, T. J. ... ... ... ... ... ... ... | Hillbury, Minehead, Somersetshire. |
| Openshaw, E. Hyde ... ... ... ... ... ... | Olveston, Gloucestershire. |
| *Ormerod, Henry ... ... ... ... ... ... ... | Westbury-on-Trym. |
| | |
| Padley, George ... ... ... ... ... ... ... ... | 2 Northampton Terrace, Swansea. |
| Page, G. Shepley ... ... ... ... ... ... ... | 78 Old Market Street, Bristol. |
| Palmer, F. Craddock, M.D. Brussels ... ... | The Corderries, Chalford. |
| *Parker, George, M.D., M.A. Cantab. ... ... | 14 Pembroke Road, Clifton. |
| Parry, J. H. ... ... ... ... ... ... ... ... ... | 99 Ashley Road, Bristol. |
| *Parson, T. Cooke ... ... ... ... ... ... ... | 21 White Ladies Road, Bristol. |
| Parsons, Francis J. C. ... ... ... ... ... | King Square, Bridgwater. |
| Parsons, Frederick ... ... ... ... ... ... | Common Hill, Frome. |
| Paterson, Donald Rose, M.D., C.M. Ed.... | 7 Newport Road, Cardiff. |
| Pauli, G. C. P. ... ... ... ... ... ... ... | 240 York Road, Bristol. |
| Pearse, W. ... ... ... ... ... ... ... ... ... | St. Tudy, Bodmin. |
| *Penny, F. ... ... ... ... ... ... ... ... ... | North Devon Infirmary, Barnstaple |
| *Penny, W. J. ... ... ... ... ... ... ... ... | 6 Westbourne Place, Clifton. |
| Pentland, Young J. ... ... ... ... ... ... | 11 Teviot Place, Edinburgh. |
| Perkins, J. H., M.D. New York ... ... ... | 14 Victoria Square, Clifton. |
| Peskett, A. W. C., M.B., B.C., M.A. Cantab. | Burnham, Somersetshire. |
| Phelps, W. Harford G., M.D. Aberd. ... | Beachfield, Weston-super-Mare. |
| Phillips, E. England ... ... ... ... ... ... | Broadwater House, Southend. |
| *Pickering, C. F. ... ... ... ... ... ... ... | 12 Berkeley Square, Bristol. |
| Pippette, Walter... ... ... ... ... ... ... | 49 Latimer Road, London, W. |
| Pizey, G. F. P.... ... ... ... ... ... ... ... | Rydal Villa, Clevedon. |
| Plaister, W. H. ... ... ... ... ... ... ... | High Road, Tottenham. |
| Pollard, Reginald, M.B. Dur. ... ... ... | Southlands, Torquay. |
| *Pountney, W. E., M.D., C.M. Ed. ... ... | 33a White Ladies Road, Bristol. |
| *Powell, J. J., M.D., Lond. ... ... ... ... | Dispensary, Bristol. |
| Powell, R. Douglas, M.D. Lond.... ... ... | 62 Wimpole Street, London, W. |
| Powell, William, M.B. Lond. ... ... ... | Hill Garden, Torquay. |
| Price, William, M.B. Lond. ... ... ... ... | 40 Park Place, Cardiff. |
| *Prichard, Arthur W.... ... ... ... ... ... | Gordon Road, Clifton. |
| *Prichard, Augustin, M.D. Berlin ... ... ... | 4 Chesterfield Place, Clifton. |
| *Prichard, J. E., M.B., B.A. Oxon. ... ... | 243 Cheltenham Road, Bristol. |
| Prichard, R., M.D., C.M. Glasg. ... ... ... | 14 Windsor Place, Cardiff. |
| Prideaux, T. Engledue P. ... ... ... ... | Wellington, Somersetshire. |
| *Pring, Arthur, B.A. Cantab. ... ... ... ... | 22 Meridian Place, Clifton. |
| Prowse, Albert P. ... ... ... ... ... ... | Yennadon, Yelverton, Devonshire. |
| *Prowse, Arthur B., M.D. Lond. ... ... ... | 5 Lansdown Place, Clifton. |
| | |
| Randolph, Charles ... ... ... ... ... ... | St. Michael's, Milverton, Somersetshire. |
| Rattray J. M., M.B., C.M., M.A. Aberd. ... | Frome. |

| | |
|---|---|
| Redwood, T. Hall, M.D., C.M., M.A. Dur. ... | The Lawn, Rhymney, Monmouthshire. |
| Rendall, John... ... ... ... ... ... ... ... | Forestside, Lymington. |
| Riddell, Andrew W. ... ... ... ... ... ... ... | St. Mary Bourne. |
| Robathan, G. B. ... ... ... ... ... ... ... ... | The Grove, Risca. |
| Robertson, Robert, M.D., C.M. Ed. ... ... | Belgrave Road, Ventnor. |
| Rolston, G. T. ... ... ... ... ... ... ... ... | 8 Osborne Villas, Stoke, Devonport. |
| *Ross, R. A. ... ... ... ... ... ... ... ... ... | Lodway Villa, Pill. |
| *Rossiter, George F., M.B. Lond. ... ... | Cairo Lodge, Weston-super-Mare. |
| *Roué, W. Barrett, M.D., M.S. Dur. ... ... | 2 Buckingham Place, Clifton. |
| Row, F. Everard ... ... ... ... ... ... ... ... | 34 Ker Street, Devonport. |
| *Roxburgh, Robert, M.B. Ed. ... ... ... ... | 1 Victoria Buildings, Weston-super-Mare. |
| Royle, John Frederick S., M.B., C.M. Aberd. | 30 Foregate Street, Worcester. |
| *Rudge, C. King ... ... ... ... ... ... ... | 145 White Ladies Road, Bristol. |
| *Rudge, H. T. ... ... ... ... ... ... ... ... ... | 5 Colston Parade, Bristol. |
| Ruffer, M. Armand, M.D., B.S., M.A. Oxon. | 27 Torrington Square, London, W.C. |
| Rundle, Edmund ... ... ... ... ... ... ... | Royal Cornwall Infirmary, Truro. |
| | |
| Scimonelli, Sig^ra. Cruce ... ... ... ... | 5 Salita dei Cattivi, Via Butera, Palermo. |
| Scott, James, M.B., C.M. Ed.... ... ... ... | 24 Eastgate Street, Stafford. |
| Scott, Richard J. H. ... ... ... ... ... ... ... | 28 Circus, Bath. |
| Searle, George C. ... ... ... ... ... ... ... | Brixham, Devonshire. |
| Searle, Richard Burford ... ... ... ... | St. Just. |
| *Semple, H. F. ... ... ... ... ... ... ... ... ... | Budleigh Salterton, Devonshire. |
| Seward, W. J., M.B. Lond. ... ... ... ... | Asylum, Colney Hatch, London, N. |
| Sewart, J. H. ... ... ... ... ... ... ... ... ... | Lostwithiel. |
| Sharpe, J. Henry, M.D., M.Ch., Roy. Univ. I. | Greenwood, Huntspill. |
| *Shaw, J. E., M.B., C.M. Ed. ... ... ... ... | 23 Caledonia Place, Clifton. |
| Sheen, A., M.D. St. And. ... ... ... ... ... | 23 Newport Road, Cardiff. |
| *Sheppard, E. J. ... ... ... ... ... ... ... | 16 Park Place, Clifton. |
| *Shorland, Walter E. ... ... ... ... ... | 189 Cheltenham Road, Bristol. |
| Shortridge, Thomas Wood, M.D. Brussels | Honiton. |
| Sincock, J. Bain ... ... ... ... ... ... ... | Friarn Street, Bridgwater. |
| Skeate, Edwin ... ... ... ... ... ... ... | 16 Paragon, Bath. |
| *Skelton, Henry, M.D. St. And. ... ... ... | Downend, Gloucestershire. |
| *Skerritt, E. Markham, M.D., B.S., B.A. Lond. | Richmond Hill, Clifton. |
| *Skinner, Stephen, M.B., C.M. Aberd. ... | Ferndale, Clevedon. |
| Smith, Frederick A. A., M.D., C.M. Glasg. | Portland House, Cheltenham. |
| *Smith, G. Munro ... ... ... ... ... ... ... | 27 All Saints Road, Clifton. |
| *Smith, J. Greig, M.B., C.M., M.A. Ab., F.R.S.E. | 16 Victoria Square, Clifton. |
| Smith, J. Sidney, M.D. St. And. ... ... ... | Bampton Street, Tiverton. |
| Smith, Noble ... ... ... ... ... ... ... ... ... | 24 Queen Anne Street, London, W. |
| *Smith, R. Shingleton, M.D., B.Sc. Lond. | Clifton Park, Clifton. |
| Smith, Samuel ... ... ... ... ... ... ... | 7 Kingsdown Parade, Bristol. |
| Smyth, Spencer T., M.D. Aberd. ... ... ... | Lansdowne Road, Bournemouth. |
| Snell, Simeon ... ... ... ... ... ... ... | 17 Eyre Street, Sheffield. |
| Society, Cardiff Medical (*Hon. Sec.*, Dr. D. R. Paterson). | |
| Society, Royal Medical and Chirurgical | 53 Berners Street, London, W. |
| *Solly, Reginald V., M.B., B.S. Lond. ... | General Hospital, Bristol. |
| Somer, James ... ... ... ... ... ... ... ... | Broadclyst. |
| *Spencer, W. H., M.D., M.A. Cantab. ... ... | 11 Warrior Square, St. Leonard's-on-Sea. |
| Spender, John K., M.D. Lond. ... ... ... | 17 Circus, Bath. |
| Stamp, W. D. ... ... ... ... ... ... ... ... ... | Ridgeway House, Plympton. |
| Stansfeld, G. M. ... ... ... ... ... ... ... ... | 19 Victoria Square, Clifton. |
| Statham, R. Whiteside ... ... ... ... ... | Cheddar, Somersetshire. |

### LIST OF SUBSCRIBERS.

| | |
|---|---|
| *Steele, Charles, M.D. Dur. ... ... ... ... | 17 Richmond Hill, Clifton. |
| Steele-Perkins, E. B. ... ... ... ... ... | 20 Sidwell Street, Exeter. |
| Stephens, Lockhart E. W. ... ... ... ... | Emsworth. |
| *Stevens, W. H. ... ... ... ... ... ... ... ... | Gloucester Road, Bristol. |
| Stewart, James, B.A. Roy. Univ. I. ... ... | Dunmurry, Stoke Bishop. |
| Stoddart, F. Wallis ... ... ... ... ... ... ... | 1 Park Street, Bristol. |
| Storry, Frederick W. ... ... ... ... ... ... | Lansdown, Stroud. |
| *Swain, James, M.D., B.S. Lond. ... ... ... | Royal Infirmary, Bristol. |
| *Swayne, J. G., M.D. Lond. ... ... ... ... | 74 Pembroke Road, Clifton. |
| *Swayne, S. H.... ... ... ... ... ... ... ... ... | 129 Pembroke Road, Clifton. |
| *Swayne, W. Carless, M.B. Lond.... ... ... | St. Paul's Road, Clifton. |
| Sydenham, George F. ... ... ... ... ... | Dulverton, Somersetshire. |
| Symonds, H. P. ... ... ... ... ... ... ... ... | 35 Beaumont Street, Oxford. |
| Syrée, A. Hugh ... ... ... ... ... ... ... ... | City of London Asylum, Stone, Dartford |
| Tait, Lawson ... ... ... ... ... ... ... ... ... | 7 The Crescent, Birmingham |
| Taylor, C. Bell, M.D. Edin. ... ... ... ... | 9 Park Row, Nottingham. |
| *Taylor, James ... ... ... ... ... ... ... ... | 36 Alma Road, Clifton. |
| Taylor, Thomas Henry ... ... ... ... ... | Thornbury, Gloucestershire. |
| Thomas, A. Garrod, M.D., C.M. Ed. ... ... | Clytha Park, Newport, Mon. |
| Thomas, Jabez ... ... ... ... ... ... ... ... | Ty Cerrig, Swansea. |
| Thomas, John T. ... ... ... ... ... ... ... ... | Chepstow Road, Newport, Mon. |
| Thomas, R. W. ... ... ... ... ... ... ... ... | Freemantle Square, Bristol. |
| Thompson, J. Tatham, M.B., C.M. Ed. ... | 23 Charles Street, Cardiff. |
| Thomson, Eustace B., M.D. C.M. Glasg.... | Albany Place, Plymouth. |
| Thomson, William, M.D., C.M. Ed. ... ... | Mustapha Superieur, Algiers. |
| *Tivy, W. J. ... ... ... ... ... ... ... ... ... | 8 Lansdown Place, Clifton. |
| Tomkins, Harding H.... ... ... ... ... ... ... | 8 Auckland Terrace, Ramsey, Isle of Man. |
| Torbock, W. H., M.D. Pennsylvania ... ... | Polruan, Cornwall. |
| Tosswill, Louis H., M.B., B.A. Cantab. ... | 28 West Southernhay, Exeter. |
| Trotter, Leslie B., M.D., C.M. Ed. ... ... | Coleford, Gloucestershire. |
| Twining, A. H. ... ... ... ... ... ... ... ... | The Knoll, Salcombe. |
| Tyley, R. P., M.D. St. And. ... ... ... ... | Wedmore. |
| Vachell, C. T., M.D. Lond. ... ... ... ... | 38 Charles Street, Cardiff. |
| Vachell, Herbert R., M.D., C.M. Aberd.... | 15 Newport Road, Cardiff. |
| Vernon, Edward ... ... ... ... ... ... ... ... | Kingston, Yeovil. |
| Wade, A. Law, M.D., B.A. Dub. ... ... ... | County Asylum, Wells, Somersetshire. |
| Wade, Reginald ... ... ... ... ... ... ... ... | Highbridge, Somersetshire. |
| *Waldo, Henry, M.D., C.M. Aberd. ... ... | 19 Pembroke Road, Clifton. |
| *Walker, Cyril H., M.B., B.A. Cantab ... ... | St. Paul's Road, Ciflton. |
| Wallace, Rev. C. Hill, M.A. Oxon. ... ... | 3 Harley Place, Clifton. |
| Walter, William Henry, M.D. Brussels ... | Ivy Villa, South Petherton. |
| Ward, J. L. W. ... ... ... ... ... ... ... ... | Victoria Street, Merthyr Tydvil. |
| *Wathen, J. Hancocke ... ... ... ... ... | 16 York Place, Clifton. |
| Watson, J. Adam ... ... ... ... ... ... ... ... | 39 Dennington Park, London, N.W. |
| Watters, George T. B., M.D., C.M. Ed. ... | Stonehouse, Gloucestershire. |
| Waugh, Alexander ... ... ... ... ... ... | Midsomer Norton. |

| | |
|---|---|
| WEATHERLY, LIONEL A., M.D., C.M. Aberd. | Bailbrook House, Bath. |
| WEBBER, WILLIAM W.... ... ... ... ... ... ... | Crewkerne. |
| WEBSTER, NORMAN P. ... ... ... ... ... ... ... | George Place, Guernsey. |
| *WEBSTER, THOMAS ... ... ... ... ... ... ... | Redland Hill, Bristol. |
| *WEDMORE, C. ERNEST, M.B., M.A. Cantab. | 11 Richmond Hill, Clifton. |
| WETHERED, FRANK J., M.D. Lond. ... ... | 34 Queen Anne Street, London, W. |
| WHISHAW, REGINALD R., M.B., B.C., B.A. Cantab. | Coombe Road, Croydon. |
| WHITFELD, W. CLARKE ... ... ... ... ... ... | 5 St. Ethelbert Street, Hereford. |
| WHYTE, ANDREW, M.D., C.M. Aberd. ... ... | Honddû House, Brecon. |
| WICKHAM, W. ... ... ... ... ... ... ... ... ... | Hill House, Tetbury. |
| WIGMORE, J., M.D. Roy. Univ. I. ... ... ... | Twerton-on-Avon. |
| *WILDING, JAMES, M.D. Dur. ... ... ... ... | Sydenham Road, Bristol. |
| WILLCOX, R. LEWIS ... ... ... ... ... ... ... | Warminster. |
| WILLETT, GEORGE G. D. ... ... ... ... ... ... | Keynsham. |
| WILLIAMS, CHARLES ... ... ... ... ... ... ... | Port Isaac. |
| WILLIAMS, D. J. ... ... ... ... ... ... ... ... | Greenfield House, Llanelly. |
| WILLIAMS, H. EGERTON ... ... ... ... ... ... | The Mount, Newport, Mon. |
| *WILLIAMS, LIONEL H. ... ... ... ... ... ... ... | Thornbury, Gloucestershire. |
| *WILLIAMS, P. WATSON, M.B. Lond. ... ... | 14 Westbourne Place, Clifton. |
| WILLIAMS, PETER ... ... ... ... ... ... ... ... | Ferryside, Carmarthenshire. |
| WILLIAMS, WILLIAM HENRY, M.D. Lond. ... | Sherborne. |
| WILSON, CLAUDE, M.D., C.M. Ed. ... ... ... | Church Road, Tunbridge Wells. |
| *WINDSOR-AUBREY, H. W. ... ... ... ... ... ... | Coronation Road, Bristol. |
| WINTERBOTHAM, L. ... ... ... ... ... ... ... | Bays Hill, Cheltenham. |
| WOOLLCOMBE, W. L. ... ... ... ... ... ... ... | 16 Lockyer Street, Plymouth. |
| | |
| YELF, LEONARD K., M.D. St. And. ... ... ... | Moreton-in-Marsh. |

### *PUBLICATIONS RECEIVED.*

From Hugo Ames, London:
*The Dwarf.* January 20th, 1891.

From Baillière, Tindall & Cox, London:
*Koch's Remedy in Throat Consumption.* By Lennox Browne. 1891.
*Aids to Sanitary Science.* By Francis J. Allan, M.D. [1890.]
*The Germ-Theory of Disease.* By D. Campbell Black, M.D. [1891.]

From Burroughs, Wellcome & Co., London:
*Desk Calendar.* 1891.
*A B C Pocket Diary.* 1891.

From Ev. E. Carreras, St. Louis:
*The Alienist and Neurologist.* January, 1891.

From Cassell & Company Limited, London:
*Hygiene and Public Health.* By B. Arthur Whitelegge, M.D. 1890.

From *El Censor*, Buenos Ayres:
*Anales de la Asistencia Pública.* No. 2. 1890.

From F. A. Davis, Philadelphia:
*The Daughter.* By William M. Capp, M.D. 1891.
*The Medical Bulletin.* January, 1891.

From Octave Dorin, Paris:
*Amygdalotomie et Hémorragie.* Par le Docteur E. J. Moure. 1891.

From Wm. J. Dornan, Philadelphia:
I. *The Prevention of the Short Leg of Hip Disease.* II. *The After-Treatment of Hip Disease.* By A. B. Judson, M.D. 1890. Reprint.

From Government Printing Office, Washington:
*Index-Catalogue of the Library of the Surgeon-General's Office, United States Army.* Vol. XI. Phædronus-Régent. 1890.

From Charles Griffin & Company, London:
*Railway Injuries.* By Herbert W. Page. 1890.

From H. K. Lewis, London:
*Nursing and Hygiene.* By R. Lawton Roberts, M.D. 1890.
*Antiseptics in Obstetric Nursing.* By John Shaw, M.D. 1890.
*Manual of Bacteriology.* By Edgar M. Crookshank, M.B. Third Edition. 1890.
*The Action of Water on Lead.* By John Henry Garrett, M.D. 1891.
*The Middlesex Hospital. Reports of the Medical, Surgical, and Pathological Registrars for the year* 1889. 1891.
*Notes on the Examination of the Sputum, Vomit, Fæces, and Urine.* By Sidney Coupland, M.D. 1891.
*The Use of the Oil of Eucalyptus Globulus in Scarlet Fever.* By J. Brendon Curgenven. 1891.
*The Thermometer in Obstetrics and Gynæcology.* By A. D. Leith Napier, M.D. 1890.

From 8 Oxford Circus Avenue, London:
*British Nurses' Association. First Annual Report.* 1889.

From Young J. Pentland, Edinburgh:
*Ophthalmoscopic Diagnosis.* By George A. Berry, M.B. 1891.
*Reports from the Laboratory of the Royal College of Physicians, Edinburgh.* Edited by J. Batty Tuke, M.D., and D. Nöel Paton, M.D. Vol. III. 1891.

From G. P. Putnam's Sons, New York:
*What is Orthopædic Surgery?* By Newton M. Shaffer, M.D. 1890.

From John Morgan Richards, London:
*Medical Reprints.* December 15th, 1890.

From Rev. T. B. Stephenson, D.D., London:
*The Children's Home.* January 15th, 1891.

From John Wright & Co., Bristol:
*Our Baby.* By Mrs. Langton Hewer. 1891.
*Lectures on Diabetes.* By Robert Saundby, M.D. 1891.

### Exchange-List.

### AFRICA.

### ALGERIA.

*Monthly.*

*Bulletin Médical de l'Algérie.* Algiers: L. Remordet et Co.

### AMERICA.

### ARGENTINE REPUBLIC.

*Monthly.*

*Anales del Circulo Médico Argentino.* Buenos Ayres: M. Biedma.

### CANADA.

*Monthly.*

*The Montreal Medical Journal.* Montreal: Gazette Printing Company.

### MEXICO.

*Twice a month.*

*Gaceta Médica.* Mexico: Avenida 2 Oriente, 726.

### PERU.

*Monthly.*

*La Crónica Médica.* Lima: Carlos Prince.

### UNITED STATES.

*Weekly.*

*The Cincinnati Lancet-Clinic.* Cincinnati: 199 West 7th Street.

*The Journal of the American Medical Association.* Chicago: 68 Wabash Avenue.

*The Medical and Surgical Reporter.* Philadelphia: N.E. Cor. 13th and Walnut Streets.

*The Medical News.* Philadelphia: Lea Brothers & Co.

*The Medical Record.* New York: William Wood & Co.

*The New York Medical Journal.* New York: D. Appleton & Co.

*Fortnightly.*

*The American Practitioner & News.* Louisville: John P. Morton and Company.

*Twice a month.*

*The Medical Age.* Detroit: George S. Davis.

*Monthly.*

*Annals of Surgery.* St. Louis: J. H. Chambers & Co.

*Index Medicus.* Detroit: George S. Davis.

*Journal of Cutaneous and Genito-Urinary Diseases.* New York: D. Appleton & Co.

*The American Journal of Ophthalmology.* St. Louis: J. H. Chambers & Co.

*The American Journal of the Medical Sciences.* Philadelphia: Lea Brothers & Co.

*The Journal of Nervous and Mental Disease.* New York: 25 West 45th Street.

*The Epitome of Medicine.* New York: G. P. Putnam's Sons.

*The Obstetric Gazette.* Cincinnati: 199 West 7th Street.

*Monthly.*

*The Saint Louis Medical and Surgical Journal.* Saint Louis: Owens Printing Company.

*The Therapeutic Gazette.* Detroit: George S. Davis.

*Yearly.*

*Annual of the Universal Medical Sciences.* Philadelphia: F. A. Davis.

*Transactions of the American Surgical Association.* Philadelphia: P. Blakiston, Son & Co.

*Occasionally.*

*Transactions of the New York Academy of Medicine.* New York: 12 West 31st Street.

### ASIA.

### CHINA.

*Quarterly.*

*The China Medical Missionary Journal.* Shanghai: Kelly & Walsh, Limited.

### INDIA.

*Monthly.*

*The Indian Medical Gazette.* Calcutta: Thacker, Spink & Co.

### JAPAN.

*Monthly.*

*The Sei-I-Kwai Medical Journal.* Tokio: Z. P. Maruya & Co.

### AUSTRALASIA.

### AUSTRALIA.

*Monthly.*

*The Australasian Medical Gazette.* Sydney: L. Bruck.

*The Australian Medical Journal.* Melbourne: Stillwell & Co.

### NEW ZEALAND.

*Quarterly.*

*The New Zealand Medical Journal.* Dunedin: J. Wilkie and Co.

### EUROPE.

### BELGIUM.

*Monthly.*

*Bulletin de l'Académie royale de médecine de Belgique.* Brussels: F. Hayez.

### BRITISH ISLES.

*Weekly.*

*British Medical Journal.* London: 429 Strand.

*The Hospital.* London: 140 Strand.

*The Lancet.* London: 234 Strand.

*Monthly.*

*The Birmingham Medical Review.* Birmingham: Hall & English.

*The Journal of Laryngology and Rhinology.* London: F. A. Davis.

*The Medical Chronicle.* Manchester: John Heywood.

*The Practitioner.* London: Macmillan & Co.

*Edinburgh Medical Journal.* Edinburgh: Oliver and Boyd.

*The Glasgow Medical Journal.* Glasgow: Alex. Macdougall.

*The Dublin Journal of Medical Science.* Dublin: Fannin & Co.

*Quarterly.*

*Archives of Surgery.* London: J. & A. Churchill.

*The Asclepiad.* London: Longmans, Green, & Co.

*The British Gynæcological Journal.* London: John Bale & Sons.

*Half-yearly.*

*The Liverpool Medico-Chirurgical Journal.* Liverpool: Medical Institution.

*The Retrospect of Medicine.* London: Simpkin, Marshall, & Co.

*Yearly.*

*Guy's Hospital Reports.* London: J. & A. Churchill.

*St. Thomas's Hospital Reports.* London: J. & A. Churchill.

*The Directory of Second-hand Booksellers.* Rochdale: James Clegg.

*The Medical Annual.* Bristol: John Wright & Co.

*The Year-Book of Treatment.* London: Cassell & Company, Limited.

*Transactions of the Royal Academy of Medicine in Ireland.* Dublin: Fannin & Co.

*Year-Book of Scientific and Learned Societies.* London: C. Griffin & Co.

DENMARK.

*Weekly.*

*Hospitals-Tidende.* Copenhagen: Jacob Lund.

FRANCE.

*Three times a week.*

*Gazette des hôpitaux.* Paris: 4 Rue de l'Odéon.

*L'Union médicale.* Paris: 11 Rue de la Grange-Batelière.

*Weekly.*

*Bulletin de l'Académie de médecine.* Paris: G. Masson.

*Le Progrès médical.* Paris: 14 Rue des Carmes.

*Twice a month.*

*Gazette de Gynécologie* Paris: O. Doin.

*Monthly.*

*Revue mensuelle de Laryngologie, d'Otologie et de Rhinologie.* Paris: 8 Place de l'Odéon.

GERMANY.

*Twice a week.*

*Deutsche Medizinal-Zeitung.* Berlin: Eugen Grosser.

*Weekly.*

*Deutsche medicinische Wochenschrift.* Berlin: George Thieme.

*Medicinische Bibliographie.* Leipzig: Breitkopf & Härtel.

*Monthly.*

*Fortschritte der Krankenpflege.* Berlin: H. Kornfeld.

*Quarterly.*

*Bibliotheca Medico-Chirurgica.* Gottingen: Vandenhoeck & Ruprecht.

GREECE.

*Weekly.*

*Γαληνός.* Athens: N. G. Passare.

HOLLAND.

*Weekly.*

*Nederlandsch Tijdschrift voor Geneeskunde.* Amsterdam: F. van Rossen.

ITALY.

*Twice a month.*

*Lo Sperimentale.* Florence: 35 Via degli Alfani.

NORWAY.

*Monthly.*

*Norsk Magazin for Lægevidenskaben.* Christiania: Th. Steens.

PORTUGAL.

*Weekly.*

*A Medicina Contemporanea.* Lisbon: José Antonio Rodrigues & Ca.

ROUMANIA.

*Twice a month.*

*Spitalul.* Bucharest: Stefan Mihalescu

RUSSIA.

*Weekly.*

*Medycyna.* Warsaw: 80 Allée de Jérusalem.

*Vrach.* St. Petersburg: C. Ricker.

SPAIN.

*Weekly.*

*El Siglo Médico.* Madrid: Magdalena, 36, 2º

*Twice a month.*

*Revista de Ciencias Médicas.* Barcelona 9 Calle Fortuny.

SWEDEN.

*Quarterly.*

*Nordiskt Medicinskt Arkiv.* Stockholm: P. A. Norstedt & Söner.

TURKEY.

*Twice a month.*

*Gazette Médicale d'Orient.* Constantinople: Edgar Whitaker.

